# Evaluating the Effectiveness and Safety of Theruptor Novo Dressing Pad in Managing Diabetic Foot Ulcer: A Prospective Study

**DOI:** 10.7759/cureus.80542

**Published:** 2025-03-13

**Authors:** Sanjay Sharma, Belehalli Pavan, Riya Gaur, Mahantesh L

**Affiliations:** 1 Wound Care, FootSecure Clinic, Bengaluru, IND

**Keywords:** chronic wound, diabetic foot ulcer, resvech score, subject satisfaction, wound dimension

## Abstract

Background: Diabetic foot ulcers are chronic complications of diabetes that frequently result in infection and may necessitate limb amputation. Despite various existing treatments like hyperbaric oxygen therapy and revascularization, a significant need remains for innovative solutions to manage wounds effectively. Theruptor novo dressing pad (Healthium Medtech Limited, India) has been designed to promote healing and potentially advance wound care. This study aims to evaluate the effectiveness and safety of the Theruptor novo dressing pad in managing Diabetic foot ulcers.

Methodology: This prospective, single-arm, multicenter study was conducted from February 2023 to March 2024 at FootSecure Clinics, Bangalore, Karnataka, India. Theruptor novo dressing pad was used, and patients were followed up for eight weeks. Patient demographics, wound features, vital signs, reduction in wound size, pain score, subject satisfaction score, and Resultados Esperados de la Cicatrización de las Heridas Crônicas (RESVECH) 2.0 score were assessed. In addition, correlation analysis between HbA1c, pain score, and ankle-brachial index (ABI) with RESVECH 2.0 score was performed.

Results: A total of 49 patients with the mean age of 59.14 ± 12.42 years were recruited. Most of the ulcers in patients were located on the foot area (n=20 (40.8%)). We observed significant reductions in wound dimensions and RESVECH 2.0 scores every week from Visit 1 to Visit 9 (p<0.05). Notably, 22 (45%) patients achieved complete healing before eight weeks of follow-up. Further, the mean pain score significantly decreased from 2.83 ± 1.59 at Visit 2 to 0.72 ± 0.88 at Visit 9 (p=0.0023), while subject satisfaction scores increased from 2.75 ± 0.78 at Visit 2 to 4.45 ± 0.91 at Visit 9 (p=0.013). Correlation analysis revealed a statistically significant and positive correlation between total RESVECH 2.0 score and pain score (r=0.392; p=0.02) at Visit 2 only.

Conclusion: Theruptor Novo dressing pad was found effective in reducing wound dimensions, improving RESVECH 2.0 scores, alleviating pain, and achieving complete wound healing.

## Introduction

Chronic lower extremity wounds are those non-healing ulcers or lesions that fail to progress through the healing process in a timely and orderly manner, typically persisting for 12 to 13 months [1,2]. These wounds occur due to underlying medical conditions such as diabetes, peripheral arterial disease, or venous insufficiency [1]. Diabetic foot ulcer is a common and highly morbid complication that occurs because of poorly managed diabetes [3]. It is defined as a breakage in the epidermal and dermal region of the foot, ankle, or leg area. Structural deformities, such as Charcot neuroarthropathy, significantly increase the risk of developing diabetic foot ulcers [4]. The World Health Organization (WHO) estimated that 433 million people were affected with diabetes in 2014, which rose to 537 million diabetic people worldwide currently [5]. Among these affected diabetic patients, approximately 19% to 34% will develop diabetic foot ulcer during their lifetime [6]. These ulcers pose a significant clinical challenge due to the high risk of infection and amputation. Notably, about 20% of the patients with diabetic foot ulcer will require lower-extremity amputation and 10% will die within one year of developing diabetic foot ulcer [7]. Thus, effective management strategies to enhance the healing process and reduce morbidity are necessitated [8].

Conventional treatment methods including vacuum-assisted closure, hyperbaric oxygen therapy, revascularization, debridement, antibiotics, and growth factors (platelet-derived growth factor and vascular endothelial growth factor) exist for chronic lower extremity wounds [9,10]. However, to address the multifactorial nature of these wounds, advanced wound care products are vital for the effective management of chronic wounds that involve innovative therapeutic approaches [11,12].

Theruptor Novo dressing pad, a novel wound care product developed by Healthium Medtech Limited, India, has been designed to promote wound healing through its unique composition and properties. This topical dressing is composed of 3-D spacer fabric made of polyethylene terephthalate (90% w/w) and polyurethane (10% w/w) + 1% w/w of dimethyl tetradecyl[3-(trimethoxysilyl)propyl] ammonium chloride (DTAC) [13]. DTAC, a cationic surfactant employs a “physical kill mechanism” to provide effective microbial protection. It functions as a physical barrier against external contaminants [13,14]. However, evidence supporting and demonstrating the efficacy of Theruptor novo dressing in treating diabetic foot ulcer is limited. Based on the above background, this prospective study aims to assess the effectiveness and safety of the Theruptor Novo dressing pad in the treatment of diabetic foot ulcer.

## Materials and methods

Study design

This was a prospective, single-arm, interventional, and multicentre study conducted between February 2023 and March 2024 to assess the effectiveness and safety of Theruptor novo dressing pad in treating diabetic foot ulcer. The patients were recruited from two different centers of FootSecure Clinic located at Sahakar Nagar and Malleshwaram in Bangalore, Karnataka, India. The study protocol was approved by the Institutional Ethical Committee (IEC), Telerad RxDx Healthcare Pvt. Ltd. Bengaluru, India. The study was registered in the Clinical Trial Registry of India (CTRI/2023/02/049571). Duly signed written informed consent was obtained from each patient before they participated in the study. The study adhered to and followed the regulations and ethical principles stated in the Declaration of Helsinki [15].

Patient recruitment and allocation

Patients with diabetic foot ulcers were screened based on the inclusion and exclusion criteria and subsequently enrolled in the study. The inclusion criteria were male and female patients aged above 18 years with ≤ 3 wounds in the lower extremity, with low to moderate exuding wounds of size ≤ 8 cm × 8 cm, with less than 50% contraction in the past four weeks even after standard treatment. Conversely, patients with wounds caused by venous or arterial insufficiency, electrical or chemical burns, wounds showing the presence of necrosis, purulence, or sinus tracts that cannot be removed by debridement, history of active Charcoat’s foot of the study foot within six months of screening, HbA1c >12%, progressive weight loss, patients undergoing treatment with corticosteroids, immunosuppressive or chemotherapeutic agents, or radiotherapy, history of other diseases which can alter the normal healing of the wound such as connective tissue disease, renal failure, liver failure, and malignancy, patients who underwent revascularization surgery in the last eight weeks, ankle-brachial index (ABI) < 0.7, and or pregnant or lactating mothers were excluded from the study.

After the screening, eligible patients received Theruptor Novo Dressing pad (Healthium Medtech Limited, India). Thereafter, patients were reviewed and followed up weekly till Week 8 (Day 56) or until their wounds healed. A window period of ±3 days was allowed for all the follow-up visits to accommodate scheduling variations. Follow-up adherence was monitored through scheduled reminders.

Data collection and follow-up

During the screening period (Visit 1, Day 0), a series of assessments such as demographics (age, gender, height, weight, body mass index (BMI), occupation, and history of smoking, alcohol, and allergy), medical history and concomitant medications, laboratory investigations (HbA1c and ABI), and clinical examination including recording of vital signs (pulse rate, temperature, systolic and diastolic blood pressure, and respiratory rate) and initial description of the wound (duration, size, and location) were recorded. The patients were then followed up for eight weeks or 56 days. During every visit of their follow-up, the percentage of wound contraction, wound pain, subject satisfaction score, and Resultados Esperados de la Cicatrización de las Heridas Crônicas (RESVECH) 2.0 score [14,16] were documented. Wound360 version 1.1 (Medtech Life, India) application was used to capture the wound photograph and dimensions.

Study endpoints

Primary Endpoints

The percentage of wound contraction was calculated using the initial (baseline) and final area every week during the follow-up visits. For the same, the dimensions of the wound, i.e., length and width were measured using a graded sterile centimeter ruler scale and captured using the wound360 version 1.1 application during all the visits. The area of the wound was then calculated by multiplying the length and width.

Secondary Endpoints

Pain score during dressing pad removal was recorded using a visual analog scale (VAS) of 10 points, from Visit 2 (Week 1) to Visit 9 (Week 8). Subject satisfaction with wound healing was assessed using a 5-point scale suggesting 5 as excellent and 1 as very poor. Lastly, wound dimensions, wound edge, type of tissue in the wound bed, the depth of the wound, exudate of the wound, Peri-wound skin, and presence of infection/inflammation were assessed using the RESVECH 2.0 scale during their eight weeks of follow-up.

Sample size estimation

Based on the epidemiology of diabetic foot ulcer in India, the prevalence was estimated at the rate of 4.5 per 1,000 populations [17]. A statistical software, G*Power version 3.1.9.2 (Heinrich Heine University Dusseldorf, Germany), was used for sample size calculation. Keeping the power at 80% and significance of 5%, and an estimated effect size of 0.5, the recruitment of 41 patients would be sufficient to study the efficacy of Theruptor dressing on wound healing. Considering a 20% dropout rate, the sample size was adjusted to 49 patients to ensure adequate statistical power.

Statistical analysis

Data analysis included descriptive and inferential statistics and was analyzed using the statistical program Graphpad v.11 (GraphPad Software, USA). Normality of the data was checked using Kolmogorov-Smirnov test, indicating normal distribution of the data. A repeated measures ANOVA was performed to compare the dependent variable across nine visits. Pearson correlation test was performed to analyze the correlation between the total RESVECH 2.0 score with the pain score, HbA1c, and ABI. The p-value of 0.05 was considered a significant cutoff.

## Results

Baseline characteristics

**Figure 1:**
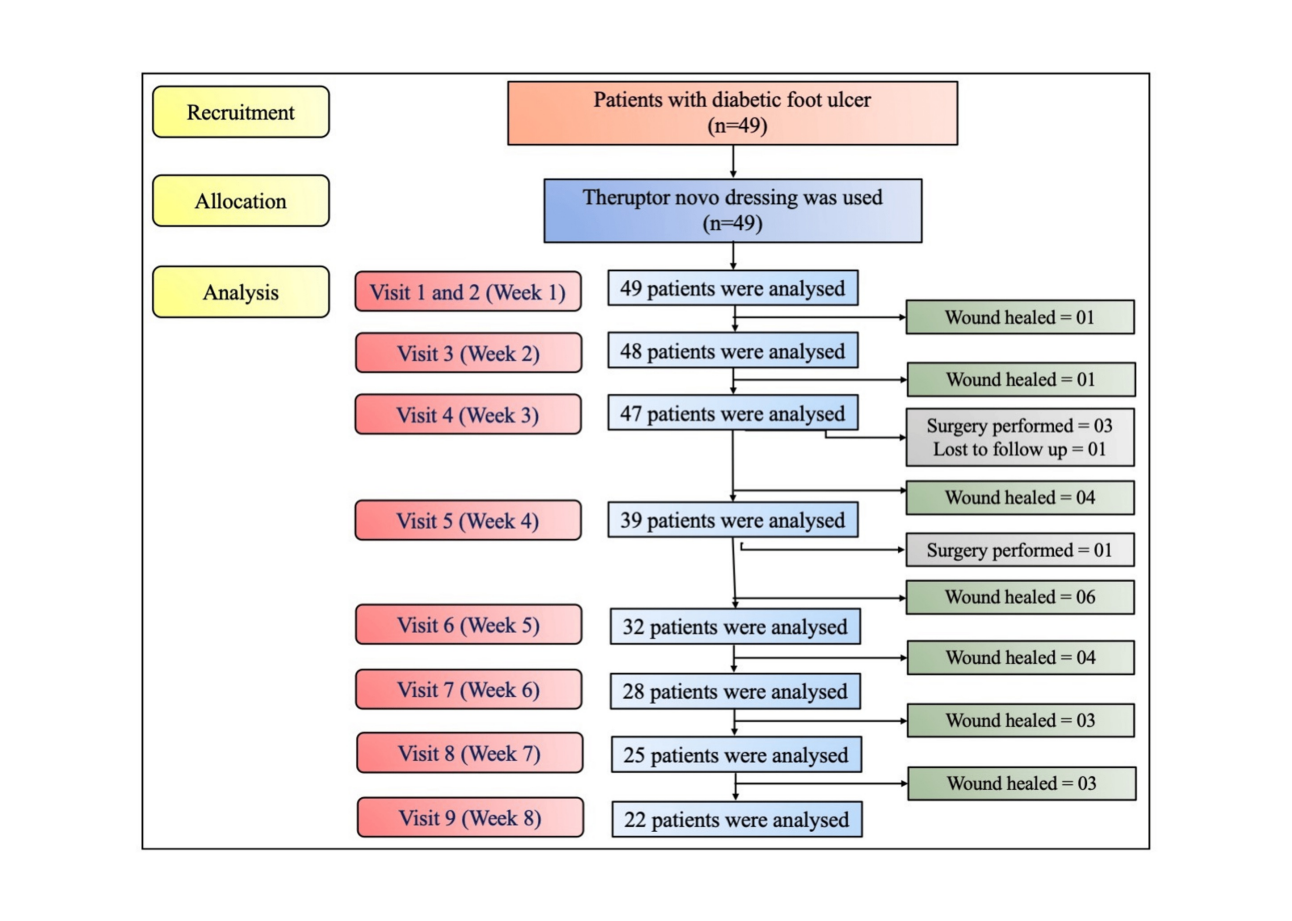
CONSORT flow chart.

The sample population was composed of a total of 49 patients recruited at FootSecure Clinic in Malleshwaram (n=33 (67.3%)) and Sahakar Nagar (n=16 (32.7%)) between February 2023 and March 2024. All patients were allocated the Theruptor Novo Dressing pad for chronic wound management and followed up till Week 8 or Day 56. The CONSORT flow diagram of patient recruitment, allocation, and analysis is shown in Figure 1.

Table 1 summarizes the baseline characteristics and vital parameters of the recruited patients. The male-to-female ratio was 39 (79.6%):10 (20.4%). The mean age of the patients was 59.14 ± 12.42 years with the youngest age reported as 30 years and the oldest as 90 years. The mean height, weight, and BMI of the patients were 164.41 ± 11.21 cm, 70.39 ± 12.21 kg, and 25.71 ± 3.95 kg/m^2^, respectively. Among 49 patients, 26 (53.1%) patients belonged to the overweight and obese category. Regarding their occupation, 33 (67.3%) patients were employed, and 16 (32.7%) patients were retired or not working. All 49 patients were diagnosed with diabetic foot ulcer. The affected foot ratio was 25 (51%) right:24 (49%) left. The most common site of the wound was foot area (n=20 (40.8%)) followed by metatarsal toes and hallux (n=19 (38.8%)). Patients had a history of hypertension (n=16 (32.7%)) and thyroid (n=4 (8.2%)). Among 49 recruited patients, 20 (40.8%) patients had normal HbA1c, and the rest had HbA1C levels of >6% while 46 (93.9%) patients had normal ABI values. All other vital parameters such as temperature, pulse rate, systolic and diastolic blood pressure, and respiratory rate were found to be normal.

**Table 1:** Demographic details, wound characteristics, and vital examination of the recruited patients. SD = standard deviation, % = percentage, cm = centimeter, kg = kilograms, pm = per minute, F = Fahrenheit, n = number of patients ABI: ankle-brachial index

Parameters	Total number of patients (n=49)
Age (years) (Mean ± SD)	59.14 ± 12.42
Gender	
Male (n (%))	39 (79.6)
Female (n (%))	10 (20.4)
Weight (kg) (Mean ± SD)	70.39 ± 12.21
Height (cm) (Mean ± SD)	164.41 ± 11.21
BMI (kg/m^2^) (Mean ± SD)	25.71 ± 3.95
Normal (18.5-24.9) (n (%))	23 (46.9)
Overweight (25-29.9) (n (%))	21 (42.9)
Obesity (30 or greater) (n (%))	05 (10.2)
Occupation	
Self-employed (n (%))	23 (46.9)
Service (n (%))	10 (20.4)
Not working (n (%))	16 (32.7)
Tobacco usage: Yes (n (%))	01 (2)
Alcohol usage: Yes (n (%))	03 (6.1)
Allergy: Yes (n (%))	01 (2)
Diagnosis: diabetic foot ulcer	49 (100)
Extremity	
Right (n (%))	25 (51)
Left (n (%))	24 (49)
Location	
Near ankle or leg (n (%))	10 (20.4)
Metatarsal (hallux and toes) (n (%))	19 (38.8)
Foot area (n (%))	20 (40.8)
HbA1c	
Normal (n (%))	20
6%-8% (n (%))	25 (51)
>8.1% (n (%))	04 (8.2)
ABI	
Normal (1-1.4) (n (%))	46 (93.9)
Abnormal (n (%))	03 (6.1)
Concomitant diseases	
Hypertension (n (%))	16 (32.7)
Thyroid (n (%))	04 (8.2)
Pulse rate (beats pm) (Mean ± SD)	80.45 ± 10.6
Temperature (F) (Mean ± SD)	98.06 ± 0.81
Systolic blood pressure (mmHg) (Mean ± SD)	131.02 ± 11.59
Diastolic blood pressure (mmHg) (Mean ± SD)	78.69 ± 7.36
Respiratory rate (breaths pm) (Mean ± SD)	19.42 ± 1.64

Study outcomes

**Figure 2:**
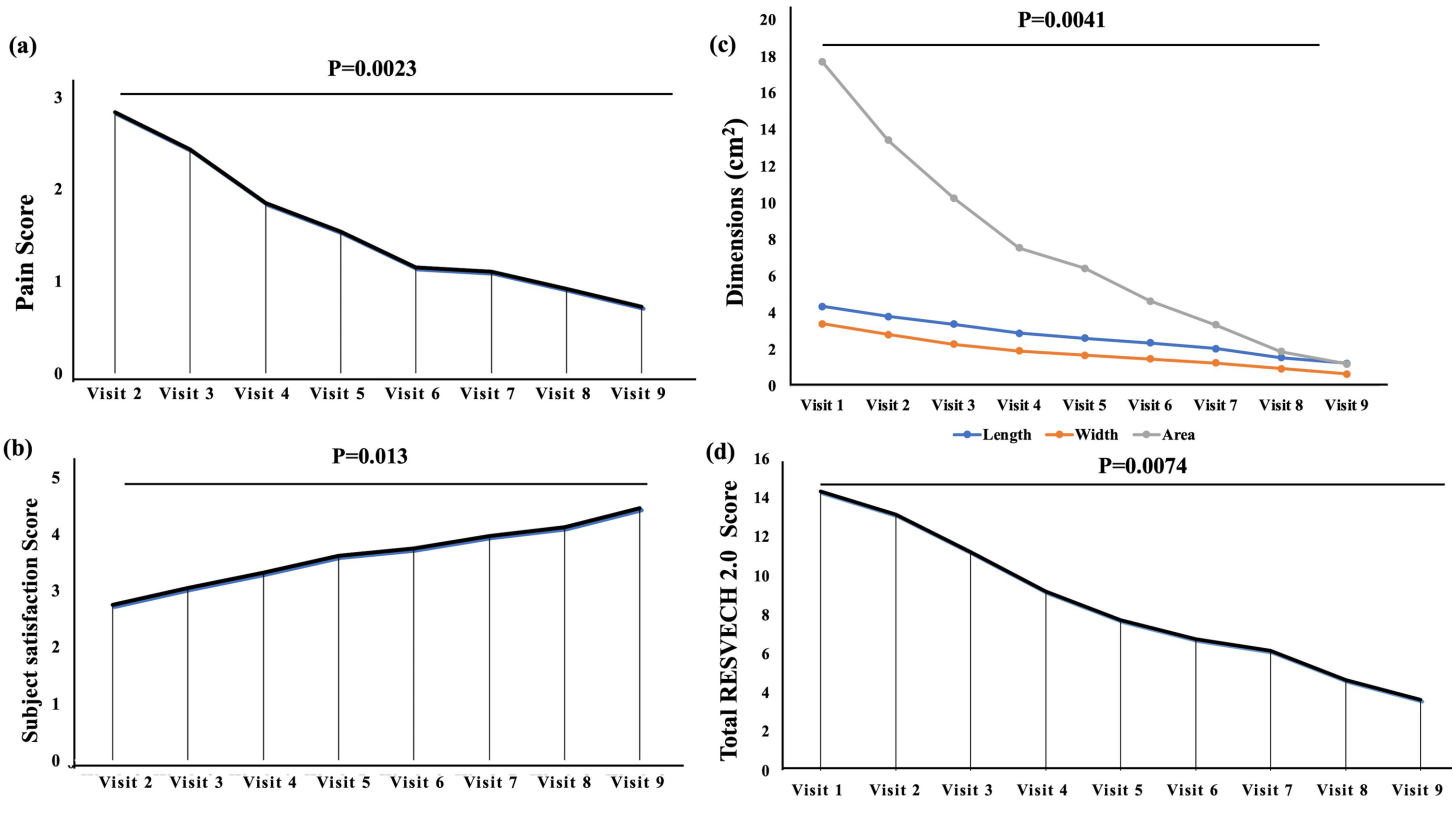
Bar with line graphs depicting primary and secondary study outcomes: (a) Pain score, (b) subject satisfaction score, (c) wound dimensions, and (d) total RESVECH 2.0 score that delineates mean with standard deviation. P-value was significant and calculated using repeated-measure ANOVA.

The primary and secondary study outcomes included wound dimensions (length, width, and area), pain score, subject satisfaction score, and total RESVECH 2.0 scores. A significant decrease was observed in pain score, wound dimensions, and total RESVECH 2.0 scores while the subject satisfaction score significantly increased during each follow-up visit (p<0.05). The mean pain score was significantly reduced from 2.83 ± 1.59 at Visit 2 to 0.72 ± 0.88 at Visit 9 (p=0.0023) (Figure 2a). Patients deduced the pain score from 0 to 5 only on a 10-point VAS scale. When the subject satisfaction score was assessed, a significant increase in the score was observed from 2.75 ± 0.78 at Visit 2 to 4.45 ± 0.91 at Visit 9 (p=0.013) (Figure 2b). Further, the mean length, width, and area of the wound were 4.35 ± 2.71 cm, 3.4 ± 1.77 cm, and 17.69 ± 17.61 cm^2^ at Visit 1 (Screening visit of Day 0), which was significantly decreased to 1.25 ± 1.25 cm, 0.67 ± 0.55 cm, and 1.21 ± 1.76 cm^2^ at Visit 9 (Week 8), respectively (p<0.05) (Figure 2c). At Visit 1, the mean total RESVECH 2.0 score for the wounds was found to be 14.32 ± 3.95, which significantly decreased to 3.63 ±3.07 at Visit 9 (p=0.0074) (Figure 2d). The pain score, subject satisfaction score, and wound dimensions recorded during each follow-up visit are summarized in Table 2.

**Table 2:** Dimensions of the wound, pain score, and subject satisfaction score. SD = standard deviation, n = number of patients, % = percentage, * = Excluded patients in whom wound has been healed, surgery performed or lost to follow up.

Wound dimension	Visit 1 (Baseline)	Visit 2 (Week 1)	Visit 3 (Week 2)	Visit 4 (Week 3)	Visit 5 (Week 4)	Visit 6 (Week 5)	Visit 7 (Week 6)	Visit 8 (Week 7)	Visit 9 (Week 8)
Patients analyzed* (n (%))	49 (100)	49 (100)	48 (98)	47 (96)	39 (79.6)	32 (65.3)	28 (57.1)	25 (51)	22 (44.9)
Wound healed till visit (n (%))	00 (0)	00 (0)	01 (2)	02 (4.1)	06 (12.2)	12 (24.5)	16 (32.7)	19 (38.8)	22 (44.9)
Wound Length, cm (Mean ± SD)	4.35 ±2.71	3.8 ±2.46	3.38 ±2.36	2.9 ± 2.2	2.62 ±2.13	2.27 ±1.73	2.05 ±1.61	1.55 ± 1.3	1.25 ±1.25
Wound Width, cm (Mean ± SD)	3.4 ± 1.77	2.81 ±1.54	2.29 ±1.44	1.92 ±1.24	1.68 ±1.28	1.49 ±0.96	1.26 ±0.79	0.95 ±0.59	0.67 ±0.55
Wound Area, cm^2^ (Mean ± SD)	17.69 ±17.61	13.43 ±14.21	10.25 ±11.93	7.54 ±8.83	6.43 ±8.19	4.63 ±5.75	3.34 ±4.66	1.88 ±2.27	1.21 ±1.76
Pain score (Mean ± SD)	-	2.83 ±1.59	2.43 ±1.59	1.85 ± 1.3	1.53 ±1.25	1.15 ±0.97	1.11 ±1.03	0.92 ±0.91	0.72 ±0.88
Subject satisfaction (Mean ± SD)	-	2.75 ±0.78	3.04 ±0.77	3.32 ±0.98	3.61 ±0.94	3.75 ±0.88	3.96 ±0.88	4.12 ±0.83	4.45 ±0.91
- 1 (n (%))	-	04 (8.2)	01 (2)	02 (4.1)	02 (4.1)	02 (4.1)	01 (2)	01 (2)	01 (2)
- 2 (n (%))	-	10 (20.4)	09 (18.4)	05 (10.2)	00 (0)	00 (0)	01 (2)	00 (0)	00 (0)
- 3 (n (%))	-	29 (59.2)	26 (53.1)	22 (44.9)	15 (30.6)	05 (10.2)	02 (4.1)	01 (2)	00 (0)
- 4 (n (%))	-	06 (12.2)	11 (22.5)	12 (24.5)	16 (32.7)	22 (44.9)	18 (36.7)	16 (32.7)	08 (16.3)
- 5 (n (%))	-	00 (0)	01 (2)	06 (12.2)	06 (12.2)	03 (6.1)	06 (12.2)	07 (14.3)	13 (26.5)

Table 3 represents the characteristics of the wounds based on RESVECH 2.0 values, detailing wound size, depth of affected tissues, condition of the edges, type of tissue in the wound bed, and levels of exudate and infection-inflammation. The lowest RESVECH 2.0 score was 5 points at Visit 1 and 0 points at Visit 9 while the highest was 24 points at Visit 1 and 10 points at Visit 9. In the infection-inflammation section of RESVECH 2.0 score, the most frequent sign was “biofilm compatible tissue,” reported in 30 (61.2%) cases, followed by “Increased pain” and “Tissue that is friable or easily bleeds,” reported in 25 (51%) cases each at Visit 1. Other reported signs were “Malodor” (n=18 (36.7%)), “Perilesional erythema” (n= 12 (24.5%)), “Increased temperature” (n=11 (22.5%)), and “Stagnant wound, not progressing” (n=11 (22.5%)). At the end of the follow-up period, zero scores were recorded for all the patients in the sub-parameters such as increased pain (compared to last dressing), perilesional erythema, perilesional edema, increased temperature, increased exudate (compared to last dressing), purulent exudate, malodor, increased wound size, and tissue pallor. No patients showed signs of satellite lesions during the study period.

**Table 3:** RESVECH 2.0 score of the patients. SD = Standard deviation, n = number of patients, % = percentage, * = Excluded patients in whom wound has been healed, surgery performed or lost to follow up. RESVECH: Resultados Esperados de la Cicatrización de las Heridas Crônicas

RESVECH 2.0 score	Value (n (%))	Visit 1 (Baseline)	Visit 2 (Week 1)	Visit 3 (Week 2)	Visit 4 (Week 3)	Visit 5 (Week 4)	Visit 6 (Week 5)	Visit 7 (Week 6)	Visit 8 (Week 7)	Visit 9 (Week 8)
Patients analysed*	-	49 (100)	49 (100)	48 (98)	47 (96)	39 (79.6)	32 (65.3)	28 (57.1)	25 (51)	22 (44.9)
Wound healed till visit	-	00 (0)	00 (0)	01 (2)	02 (4.1)	06 (12.2)	12 (24.5)	16 (32.7)	19 (38.8)	22 (44.9)
Dimension of the lesion	0 cm^2^	00 (0)	01 (2)	01 (2)	03 (6.1)	04 (8.2)	03 (6.1)	01 (2)	03 (6.1)	06 (12.2)
-	<4 cm^2^	06 (12.2)	12 (24.5)	20 (40.8)	21 (42.9)	19 (38.8)	18 (36.7)	21 (42.9)	19 (38.8)	14 (28.6)
-	4 < 16 cm^2^	24 (49)	19 (38.8)	13 (26.5)	13 (26.5)	08 (16.3)	09 (18.4)	05 (10.2)	03 (6.1)	02 (4.1)
-	16 < 36 cm^2^	11 (22.5)	11 (22.5)	09 (18.4)	09 (18.4)	07 (14.3)	02 (4.1)	01 (2)	00 (0)	00 (0)
-	36 < 64 cm^2^	08 (16.3)	06 (12.2)	05 (10.2)	01 (2)	00 (0)	00 (0)	00 (0)	00 (0)	00 (0)
Depth/tissues affected	Healed intact skin	00 (0)	01 (2)	03 (6.1)	07 (14.3)	06 (12.3)	06 (12.3)	04 (8.2)	05 (10.2)	10 (20.4)
-	Compromise of the dermis -epidermis	09 (18.4)	14 (28.6)	14 (28.6)	20 (40.8)	21 (42.9)	20 (40.8)	22 (44.9)	18 (36.7)	11 (22.5)
-	Compromise of subcutaneous tissue	27 (55.1)	24 (49)	26 (53.1)	16 (32.7)	10 (20.4)	05 (10.2)	02 (4.1)	02 (4.1)	01 (2)
-	Muscle impairment	07 (14.3)	07 (14.3)	03 (6.1)	04 (8.2)	02 (4.1)	01 (2)	00 (0)	00 (0)	00 (0)
-	Compromise of bone and/or adjacent tissues	06 (12.2)	03 (6.1)	02 (4.1)	00 (0)	00 (0)	00 (0)	00 (0)	00 (0)	00 (0)
Edges	Not distinguishable	00 (0)	01 (2)	03 (6.1)	05 (10.2)	04 (8.2)	03 (6.1)	04 (8.2)	08 (16.3)	07 (14.3)
-	Diffuse	04 (8.2)	03 (6.1)	04 (8.2)	04 (8.2)	08 (16.3)	08 (16.3)	06 (12.2)	07 (14.3)	05 (10.2)
-	Delimited	24 (49)	24 (49)	26 (53.1)	32 (65.3)	23 (46.9)	20 (40.8)	17 (34.5)	10 (20.4)	10 (20.4)
-	Damaged/ deteriorated	20 (40.8)	19 (38.8)	13 (26.5)	05 (10.2)	02 (4.1)	01 (2)	01 (2)	00 (0)	00 (0)
-	Thickened	01 (2)	02 (4.1)	02 (4.1)	01 (2)	02 (4.1)	00 (0)	00 (0)	00 (0)	00 (0)
Type of tissue in wound bed	Necrotic	04 (8.2)	03 (6.1)	00 (0)	00 (0)	00 (0)	00 (0)	00 (0)	00 (0)	00 (0)
-	Necrotic tissue and/or slough in the bed	25 (51)	19 (38.8)	13 (26.5)	06 (12.2)	03 (6.1)	01 (2)	00 (0)	00 (0)	00 (0)
-	Granulation tissue	15 (30.6)	21 (42.9)	25 (51)	27 (55.1)	18 (36.7)	12 (24.5)	07 (14.3)	01 (2)	01 (2)
-	Epithelial tissue	05 (10.2)	05 (10.2)	07 (14.3)	09 (18.4)	13 (26.5)	14 (28.6)	17 (34.7)	17 (34.7)	12 (24.5)
-	Closed/ healing	00 (0)	01 (2)	03 (6.1)	05 (10.2)	05 (10.2)	05 (10.2)	04 (8.2)	07 (14.3)	09 (18.4)
Exudate	Dry/ With exudate leakage	11 (22.5)	04 (8.2)	01 (2)	01 (2)	01 (2)	02 (4.1)	01 (2)	01 (2)	00 (0)
-	Humid	05 (10.2)	07 (14.3)	13 (26.5)	14 (28.6)	16 (32.7)	11 (22.5)	13 (26.5)	18 (36.7)	19 (38.8)
-	Wet	15 (30.6)	15 (30.6)	16 (32.7)	18 (36.7)	16 (32.7)	18 (36.7)	13 (26.5)	05 (10.2)	03 (6.1)
-	Saturated	18 (36.7)	23 (46.9)	18 (36.7)	14 (28.6)	06 (12.3)	01 (2)	01 (2)	01 (2)	00 (0)
Infection/ Inflammation	Increased pain	25 (51)	20 (40.8)	16 (32.7)	11 (22.5)	07 (14.3)	04 (8.2)	00 (0)	00 (0)	00 (0)
-	Perilesional erythema	12 (24.5)	09 (18.4)	07 (14.3)	05 (10.2)	01 (2)	00 (0)	00 (0)	00 (0)	00 (0)
-	Perilesional edema	06 (12.3)	10 (20.4)	05 (10.2)	03 (6.1)	01 (2)	01 (2)	01 (2)	00 (0)	00 (0)
-	Increased temperature	11 (22.5)	10 (20.4)	08 (16.3)	04 (8.2)	01 (2)	00 (0)	00 (0)	00 (0)	00 (0)
-	Increased exudate	07 (14.3)	05 (10.2)	03 (6.1)	01 (2)	00 (0)	00 (0)	00 (0)	00 (0)	00 (0)
-	Purulent exudate	07 (14.3)	07 (14.3)	06 (12.3)	06 (12.3)	02 (4.1)	01 (2)	01 (2)	00 (0)	00 (0)
-	Tissue that is friable or easily bleeds	25 (51)	24 (49)	23 (46.9)	19 (38.8)	11 (22.5)	07 (14.3)	07 (14.3)	06 (12.3)	04 (8.2)
-	Stagnant wound, not progressing	11 (22.5)	10 (20.4)	13 (26.5)	07 (14.3)	06 (12.3)	04 (8.2)	02 (4.1)	02 (4.1)	01 (2)
-	Biofilm compatible tissue	30 (61.2)	28 (57.1)	21 (42.9)	12 (24.5)	06 (12.3)	02 (4.1)	02 (4.1)	02 (4.1)	01 (2)
-	Malodor	18 (36.7)	16 (32.7)	11 (22.5)	08 (16.3)	05 (10.2)	02 (4.1)	02 (4.1)	00 (0)	00 (0)
-	Hypergranulation	01 (2)	01 (2)	02 (4.1)	03 (6.1)	05 (10.2)	04 (8.2)	05 (10.2)	03 (6.1)	02 (4.1)
-	Increased wound size	01 (2)	01 (2)	01 (2)	00 (0)	01 (2)	00 (0)	00 (0)	00 (0)	00 (0)
-	Satellite lesions	00 (0)	00 (0)	00 (0)	00 (0)	00 (0)	00 (0)	00 (0)	00 (0)	00 (0)
-	Tissue pallor	01 (2)	02 (4.1)	01 (2)	01 (2)	00 (0)	00 (0)	00 (0)	00 (0)	00 (0)
Total score	(Mean ± SD)	14.32 ± 3.95	13.14 ± 4.67	11.23± 4.57	9.21 ±4.2	7.74 ±3.87	6.75 ±3.22	6.14 ±2.66	4.64 ±2.73	3.63 ± 3.07

**Figure 3:**
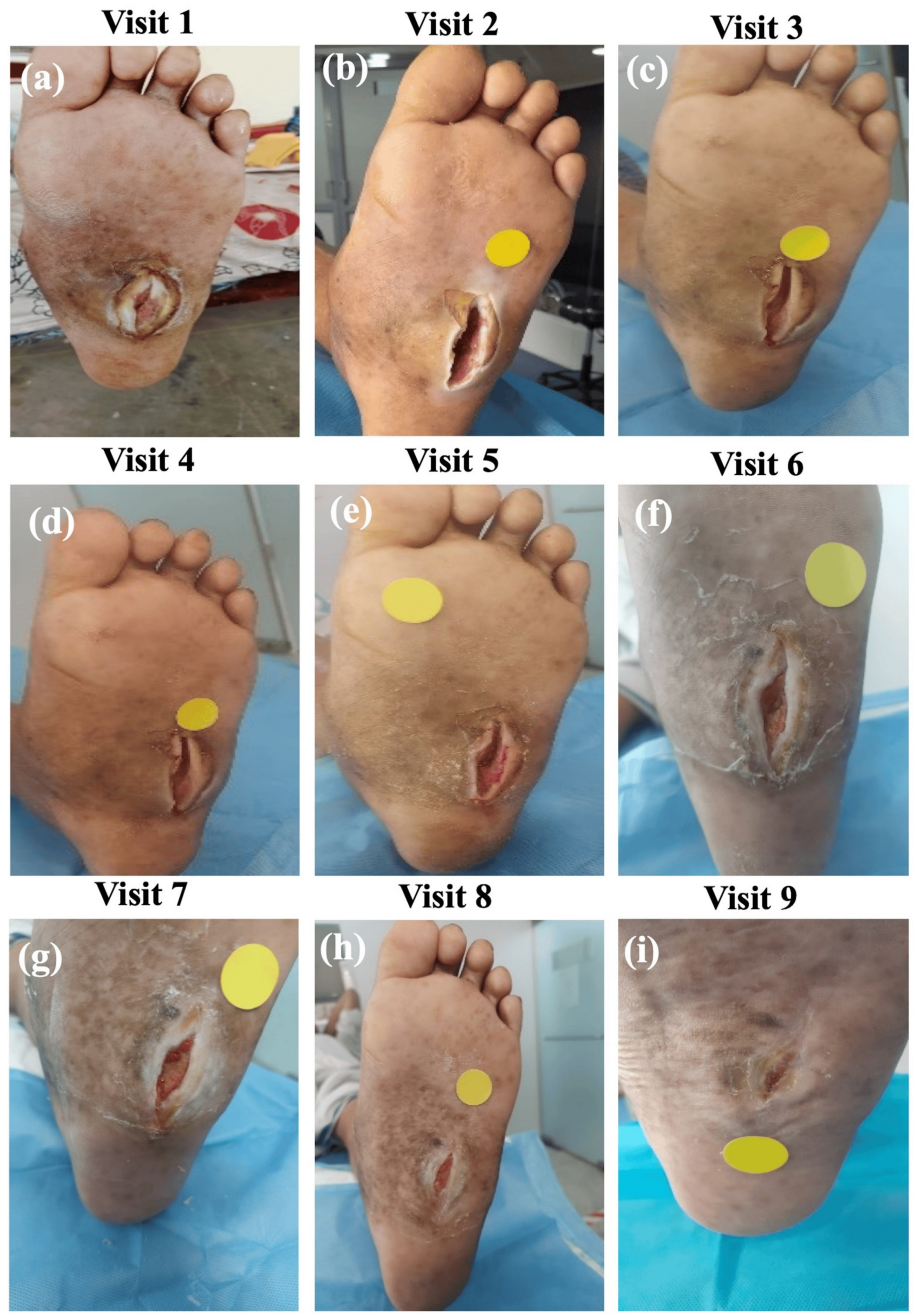
Representative images of a patient wound at (a) Visit 1, (b) Visit 2, (c) Visit 3, (d) Visit 4, (e) Visit 5, (f) Visit 6, (g) Visit 7, (h) Visit 8, and (i) Visit 9 (healed wound).

Additionally, wound images of each patient were captured using the Wound360 application during every follow-up visit to assess the wound healing progress. Figures 3a-3i present representative images of wound healing in a patient over the follow-up period. Complete wound healing was achieved in one (2%), two (4.1%), six (12.2%), 12 (24.5%), 16 (32.7%), 19 (38.8%), and 22 (44.9%) patients during Visit 3 (Week 2), 4 (Week 3), 5 (Week 4), 6 (Week 5), 7 (Week 6), 8 (Week 7), and 9 (Week 8), respectively. Notably, no adverse events were observed in any of the patients during the follow-up of eight weeks.

Inferential analysis

Lastly, we analyzed the correlation of HbA1c, pain score, and ABI with RESVECH 2.0 score using Pearson's correlation test. Among these, we found a statistically significant and positive correlation between total RESVECH 2.0 score and pain score (r=0.392; p=0.02) at Visit 2 only. Based on the categorization of Hinkle et al., the data indicate a moderate positive correlation suggesting a higher RESVECH 2.0 score is associated with a high pain score [18]. However, no statistically significant dependence was observed between total RESVECH 2.0 score with HbA1c (r=0.19, p=0.191), and ABI (r=0.22, p=0.129) at baseline.

## Discussion

In the present study, the effectiveness and safety of the Theruptor novo dressing pad for the treatment of diabetic foot ulcer were evaluated, demonstrating promising results. Our findings showed that Theruptor novo dressing pad significantly increases the wound healing process and improves the overall quality of life of patients with diabetic foot ulcer. The advanced technology of Theruptor novo dressing including antimicrobial properties and moisture-managing materials contributes to its efficacy, providing new insights into the wound care management strategies in healthcare settings.

The prevalence of foot ulcers is notably higher among males in all age groups, particularly elderly people [19]. In our study, the patient population comprised of 49 patients with the predominance of male category and a mean age of 59.14 years, which reflects a common demographic profile observed in chronic lower extremity wounds studies [19-21]. In a prospective study, Cazzell et al. assessed the efficacy of acellular dermal matrix in patients with complex chronic lower extremity wounds with deep exposure. The authors found that 70% of the recruited patients were males and reported a mean age of 56.5 years, which is in concordance with our study [21]. Additionally, a notable proportion of patients were overweight or obese, highlighting the association between metabolic factors and the development of diabetic foot ulcers.

Notably, the total study follow-up period was 56 ± 3 days, which was based on the outcomes of diabetic foot ulcers studies. The duration of the wound healing process typically relies on the size and dimensions of the wound ulcers [22]. Large and complex ulcers require more time to heal [23]. In our study, the wound size varied from 1 cm^2^ to 64 cm^2^ initially. Despite the large sizes of ulcers, wound healing was achieved in 45% of the patients (n=22/49) before the timeframe of eight weeks, highlighting the effectiveness of the treatment protocol employed. In a study, Hahn et al. reported a mean ulcer area of 16.3 cm^2^, which is similar to our data with a mean score of 17.69 cm^2^ [24]. When the wound dimensions over the nine visits period were measured, a significant reduction in length, width, and area of the ulcer was found every week, indicating progressive wound healing (p<0.05). The data suggest the importance of noting the size and area of the wound before considering treatment options for diabetic foot ulcer. 

Unhealed foot ulcer may lead to infections and amputation, thus deteriorating the health condition of the patient and significantly impacting the quality of life [25,26]. Theruptor novo is an anti-microbial dressing that is based on a “physical kill mechanism” and protects the wound from microbial contamination. Notably, no infection or adverse events were reported at any period of the study. Studies in the literature suggest that patients with diabetic foot ulcer suffer amputation with a prevalence rate of 6.5% to 18.5% [21,27]. However, only two patients in our study went for amputation surgery. The amputation rate was only 4%, which is less than the prevalent amputation rate, suggesting the efficacy of Theruptor novo dressing in the treatment of diabetic foot ulcer.

Nowadays, several instruments are commonly being used for the measurement of risk assessment, wound-related quality of life, or wound healing in diabetic foot ulcer. RESVECH score is a useful nine-item tool that measures the wound healing process. RESVECH 1.0 was created in 2010; however, RESVECH 2.0 scale is currently in use. It is composed of a six-dimension Likert-type scale, ranging from 0 to 35 [27-29]. The six dimensions comprise wound area, depth, edges, type of tissue in the wound bed, exudate, and infection/inflammation. A zero score denotes complete wound healing [29]. In our study, zero RESVECH 2.0 score was achieved in eight patients at the end of the study. Initially, the mean total RESVECH 2.0 score of the recruited patient was 14.3, which is similar to the score observed by Rodrigues et al. in a European Portuguese population. Further, we observed a significant reduction in the total score every week, suggesting a significant improvement in the wound condition of the patient [30].

In addition, we evaluated pain scores and subject satisfaction. A significant reduction in pain score from 2.83 ± 1.59 at Visit 2 to 0.72 ±0.88 at Visit 9 and increases in satisfaction levels from 2.75 ± 0.78 at Visit 2 to 4.45 ± 0.91 at Visit 9 were observed over the study period, indicating improvements in physical healing, patients' subjective experiences, and quality of life. Zero pain score was attained in 20 patients at the end of the study. Furthermore, inferential analysis provided insights into the associations between various variables. A positive and moderate correlation between RESVECH 2.0 score and pain score suggests that higher wound severity correlates with increased pain levels. However, no significant dependence was found between RESVECH 2.0 score and HbA1c or ABI, indicating that wound severity may not be directly influenced by glycemic control or vascular status.

Limitations

Lack of a control group was one of the major limitations of this study. While the efficacy of the Theruptor Novo Dressing Pad was demonstrated through significant reductions in wound area and RESVECH 2.0 scores, the absence of a control group prevented direct comparison with the standard of care. Although results could be compared with findings from existing literature, however, it would not be ideal due to variations in study designs and patient populations. Another major limitation was a shorter follow-up duration of eight weeks, which is an insufficient length of time for large ulcers to heal, as complete closure was observed in 45% of patients. Additionally, the study may be subject to selection bias in patient recruitment due to single-arm study design. This may limit the generalizability of the findings to broader patient populations. During the study, two patients were withdrawn due to amputation, two other patients due to skin grafting surgeries, and one patient was lost to follow-up. 

## Conclusions

The present study evaluated the effectiveness and safety of the Theruptor Novo Dressing Pad in managing diabetic foot ulcers over eight weeks. Theruptor novo dressing pad showed healing ability by reducing the size of large and chronic lower extremity wounds. The amputation risk was significantly low. Overall results of this study demonstrated the efficacy of Theruptor novo dressing pad to treat diabetic foot ulcers by reducing wound dimensions, improving RESVECH 2.0 scores, alleviating pain, and promoting wound closure. Notably, no adverse events or complications were reported, indicating the safety profile of the dressing. Theruptor Novo Dressing Pad was found to be effective and well-tolerated in the management of diabetic wound ulcers. Thus, Theruptor Novo may be considered a standard care option for these challenging wounds. Future research with larger sample sizes and comparative studies could further validate these findings and optimize treatment protocols for broader clinical applications.
